# Exposure to total and methylmercury among pregnant women in Suriname: sources and public health implications

**DOI:** 10.1038/s41370-020-0233-3

**Published:** 2020-05-28

**Authors:** Jeffrey K. Wickliffe, Maureen Y. Lichtveld, C. Wilco Zijlmans, Sigrid MacDonald-Ottevanger, Martin Shafer, Christa Dahman, Emily W. Harville, Stacy Drury, Gwendolyn Landburg, Paul Ouboter

**Affiliations:** 1 Department of Global Environmental Health Sciences, School of Public Health and Tropical Medicine, Tulane University, New Orleans, LA 70112, USA; 2 Academic Hospital Paramaribo, Scientific Research Center Suriname, Paramaribo, Suriname; 3 Faculty of Medical Sciences, Anton de Kom University of Suriname, Paramaribo, Suriname; 4 Wisconsin State Laboratory of Hygiene, University of Wisconsin–Madison, Madison, WI 53718, USA; 5 Department of Epidemiology, School of Public Health and Tropical Medicine, Tulane University, New Orleans, LA 70112, USA; 6 Department of Psychiatry and Behavioral Sciences, School of Medicine, Tulane University, New Orleans, LA 70112, USA; 7 National Zoological Collection of Suriname/Environmental Research Center (NZCS/CMO), Anton de Kom University of Suriname, Paramaribo, Suriname; 8 Institute for Neotropical Wildlife and Environmental Studies (NeoWild), Paramaribo, Suriname

**Keywords:** Mercury, Methylmercury, Gold mining, Fish, Neurodevelopment

## Abstract

Previous research has found that women and children living in rural, interior communities in Suriname have high concentrations of mercury in hair. Freshwater fish from these areas also have high concentrations of mercury. Artisanal and small-scale gold mining operations in parts of the country use elemental mercury to extract gold from soils and sediments. Total mercury and methylmercury concentrations have been determined in hair and blood from pregnant women across the country. Pregnant women from interior communities have significantly higher concentrations of both total and methylmercury in hair (median total mercury in hair 3.64 μg/g) compared with pregnant women from two urban coastal cities, Paramaribo (0.63 μg/g) and Nickerie (0.74 μg/g). Total and methylmercury concentrations in blood and hair are highly correlated (*r*=0.986, *r*=0.974) with methylmercury making up 86% of the total in blood and 97% of the total in hair. Most women in the interior regions rely heavily on local fish as part of their regular diet, and many live outsides of areas with active ASGM operations. This study demonstrates that diet and fish consumption largely govern mercury exposures in pregnant women in Suriname.

## Introduction

Exposure to mercury has long been and continues to be a public health problem [1, 2]. The primary source of exposure to mercury in the general population worldwide is through fish consumption [1, 3-16]. While fish is an excellent, low-fat source of nutrients including essential elements (e.g., selenium, iodine), polyunsaturated fatty acids (e.g., omega-3 fatty acids), and protein, mercury in fish, especially methylmercury in high concentrations, can negatively impact both physical development and the neurological system [1-3, 5, 17]. Methylmercury is the predominant chemical form of mercury in fish [1, 2, 15]. Several studies have documented the negative developmental neuropsychological effects that methylmercury has especially for those exposed pre- and peri-natally [3, 18-22]. The study by Marsh et al. (1997) and the cohort studies in the Faroe Islands and New Zealand have been used by health agencies such as the USEPA and WHO to develop reference values and regulatory limits to protect human health [23-25]. The USEPA has established a reference dose (RfD) for chronic exposure to methylmercury of 1E – 4 mg/kg/day designed to protect neurodevelopment during gestation [23, 25]. Under simplifying deterministic assumptions, this oral daily dose corresponds to a blood methylmercury concentration of 5.8 μg/l and a hair total mercury concentration of ~1.1 μg/g with the latter being used as an action level by the USEPA. Methylmercury at a concentration of 5.8 μg/l in cord blood is also used as an action level by the USEPA and USCDC and is estimated to correspond to a maternal blood concentration of 3.5 μg/l [26-28]. The WHO has also established a provisional tolerable daily intake of methylmercury of 2.3E – 4 mg/kg/day, which is similar to the USEPA’s RfD [24]. Using a fish consumption rate of 0.0175 kg/day, the USEPA has established a maximum tissue concentration of methylmercury in fish of 0.3 mg/kg [25]. The WHO has established a maximum tissue concentration of methylmercury in fish of 0.5 mg/kg, which is again similar to the USEPA’s tissue criterion [24].

Currently, anthropogenic sources account for about 30% of the annual emissions of mercury to air [29]. Another 10% is natural in origin with the remaining 60% coming from reemissions of previously released mercury which is thought to derive mostly from anthropogenic emissions over the last 200 years [29]. Human activities are believed to have increased total atmospheric mercury concentrations by ~450% over natural levels [15]. Anthropogenic sources of mercury can also be quite varied [2, 30-33]. In many areas, a major contributing source of mercury is artisanal and small-scale gold mining (ASGM) activity, which is estimated to account for about 38% of total global anthropogenic emissions [15, 34-40]. In many ASGM areas, it remains a common practice to use elemental mercury in the gold extraction/amalgamation process, and an appreciable amount of the mercury is subsequently lost to the environment [6, 34-44]. Elemental mercury volatilizes to the atmosphere where photochemical reactions convert it to inorganic mercury which can then be redeposited through wet or dry deposition. Both forms can be converted to methylmercury through reducing reactions carried out by anaerobic bacteria in soils and sediments. Unlike elemental and inorganic forms of mercury, methylmercury readily enters the food chain and tends to accumulate in predatory fish, especially large piscivorous fish that often serve as a primary source of dietary protein for communities [43].

In the Republic of Suriname, South America, there are active ASGM operations in the eastern and southeastern part of the country [6, 8, 42-44]. High concentrations of mercury have been found in freshwater fish collected from active ASGM areas [6, 8, 43, 44]. There is growing concern that residents in these interior regions of Suriname, including those downwind from ASGM sites, may be at increased risk of exposure to unsafe concentrations of mercury in fish, which in some areas is their only reliable source of dietary protein [6, 8, 43-45].

This study builds on an initial biomarker project examining concentrations of total mercury in hair samples from a cohort of women and children in a number of interior villages in Suriname [6, 8, 46]. Results from that work indicate that residents in many of the interior villages near ASGM operations or downwind from such activities are indeed exposed to high levels of mercury [6, 8, 46]. As reported herein, continued biomonitoring of pregnant women in Suriname supports these previous findings, with high concentrations of total mercury observed in hair of women from interior sites. In contrast, substantially lower concentrations are observed in hair from pregnant women from two coastal cities, Paramaribo and Nickerie. This suggests there are distinct and region-specific sources of exposure to mercury where closer monitoring of levels and efforts to abate deposition of mercury into the environment and food chain should be directed. To date, only concentrations of total mercury in hair have been determined, making more definitive sourcing of exposure difficult. As an exposure biomarker, total mercury in hair and blood from humans is mostly methylmercury (usually averaging between 60 and 90% of the total), especially in the general population that is not exposed occupationally or through some non-dietary source (e.g., tainted personal care products) to elemental or inorganic mercury [2, 26-28, 47]. Chemically distinguishing both total mercury and methylmercury in biospecimens such as hair and blood provides a means by which sources of exposure can be more specifically identified [47]. Based on previous research and knowledge of the dietary habits of these communities, it is hypothesized that total mercury concentrations in hair and blood from pregnant women in the interior of Suriname will be elevated and that most of the total mercury in hair and blood will be methylmercury.

## Materials/subjects and methods

**Fig. 1:**
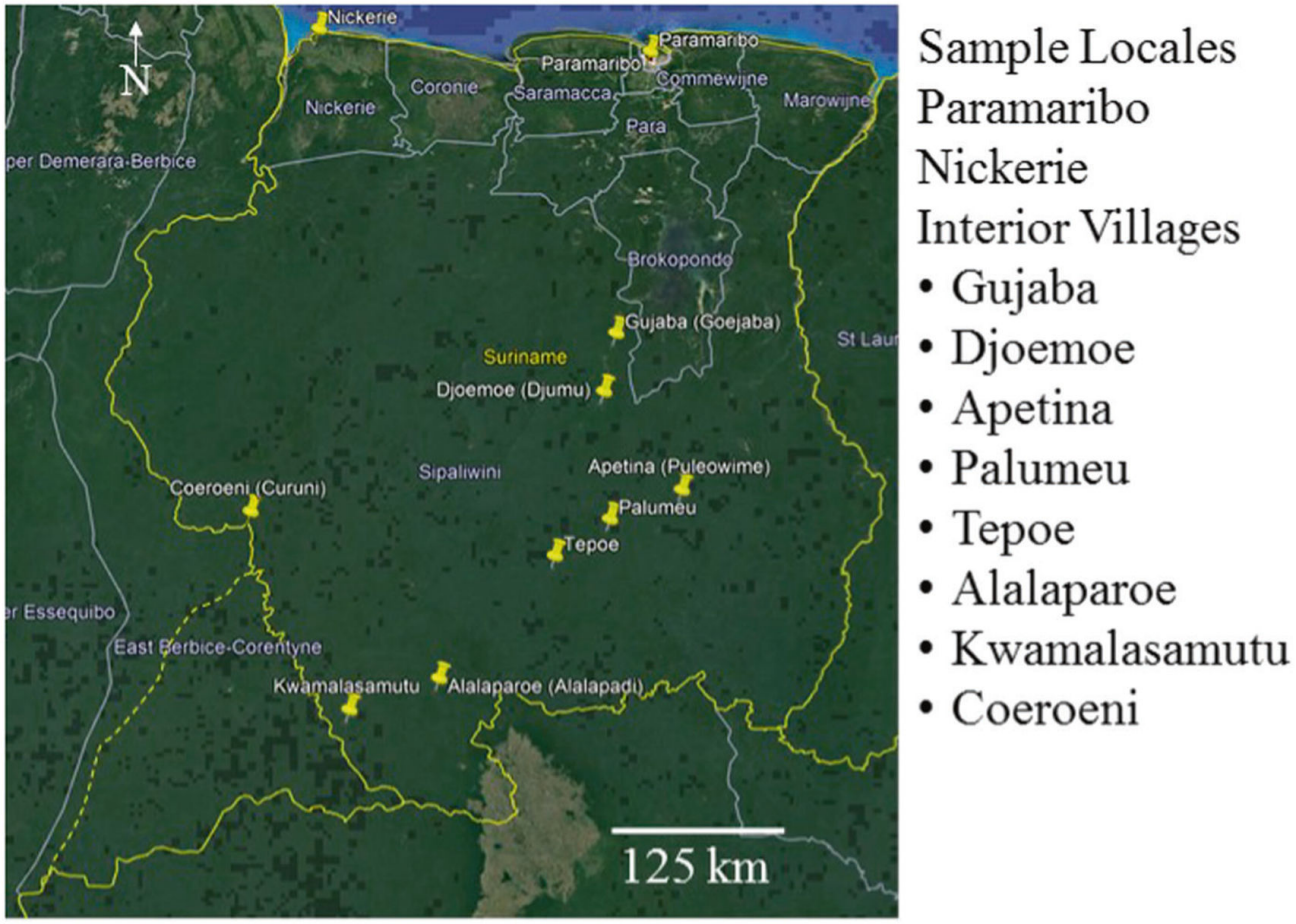
Sites where human subjects (pregnant volunteers) have been recruited for the CCREOH biomonitoring and health evaluation research project. Village names are provided for the sites in the interior.

As part of the Caribbean Consortium of Research in Environmental and Occupational Health research program, 1000 pregnant women and their babies have been recruited from both coastal and interior sites (Fig. 1) [48]. Inclusion criteria were that women, ages 16–45, were pregnant and in their first or second trimester. A primary goal of this environmental epidemiological study is to examine the impact of exposure to Hg, other heavy metals, and pesticides during pregnancy and early childhood on pediatric neurodevelopment. Samples of hair, whole blood, serum, urine, and buccal cells are being collected from each participant and cord blood is collected at birth. Neurodevelopment in children will be assessed at 12, 24, 36, and 48 months of age. This study has been approved by the Institutional Review Board at Tulane University and the Human Subjects Protection Board in Suriname. All participants provided written informed consent prior to being involved in this research project. Thus far, total mercury concentrations in hair have been determined for 505 pregnant women from Paramaribo, 172 pregnant women from Nickerie, and 130 women from the interior. Sample sizes were selected based on power analyses designed to detect differences among mercury exposures on a regional scale as indicated previously. Total mercury concentrations were determined using cold vapor atomic absorption spectroscopy (CVAAS) as reported previously [8, 46, 49].

To more conclusively identify the source(s) of exposure to mercury, hair, blood, and urine from an additional 75 pregnant women, 20 from Paramaribo, 20 from Nickerie, and 35 from interior sites were analyzed for both total and methylmercury (Fig. 1). Women in the interior were from Gujaba (*n* = 12), Djoemoe (*n* = 3), Palumeu (*n* = 8), Coeroeni (*n* = 1), Tepoe (*n* = 6), Apetina (*n* = 2), Kwamalasamutu (*n* = 1), and Alalaparoe (*n* = 2). Gujaba and Djoemoe are near current ASGM sites while Apetina is ~50 km from the nearest ASGM activity. There are no current ASGM activities in proximity to the other sites that we are aware of. Samples sizes were selected based on previous results in women’s hair samples based on regional data. This study was not designed to examine differences among interior villages which is part of an ongoing research project. Samples were shipped frozen on dry ice to the University of Wisconsin–Madison State Laboratory of Hygiene Trace Element Research Laboratory using a formal chain-of-custody process. Concentrations of total and methylmercury were determined by cold vapor atomic fluorescence spectrometry (CVAFS) after standard digestion and distillation protocols [49-55]. Details are provided below including results from analysis of exogenous vs endogenous total mercury in hair samples (Supplementary Table S1). Standard reference materials, matrix spikes, and method duplicates were used for quality assurance and quality control (QA/QC). QA/QC indicators are provided in Supplementary tables (Tables S2-S4).

Total mercury (HgT) in whole blood was determined by ICP-MS following the HgT biomonitoring method developed by the CDC [56]. A sub-sample of blood is removed from the collection vial after thorough homogenization of the collected blood by vortexing, and diluted (1 part sample + 1 part water + 48 parts diluent) for ICP-MS analysis. The diluent contains tetramethylammonium hydroxide (TMAH) and Triton X-100 to solubilize blood components and prevent deposition to the sample introduction system, as well as ammonium pyrrolidine dithiocarbamate (APDC) and ethyl alcohol to stabilize the metals and blood components. The internal standards, rhodium, iridium, and tellurium are added to all blood and QC samples to correct for variations in instrument response (drift and sample matrix differences).

Total mercury (HgT) in urine was determined by ICP-MS following the HgT biomonitoring method developed by the CDC [57]. A sub-sample of urine is removed from the collection tube after thawing and careful homogenization of the urine in the collection tube by vortexing, and diluted (1 part sample + 1 part water + 8 parts diluent) for ICP-MS analysis. Similar to the blood method, the diluent contains TMAH and Triton X-100 to solubilize organic components of the urine and prevent deposition to the sample introduction system, as well as APDC and ethyl alcohol to stabilize the metals and urine organics. Rhenium is added as the internal standard.

Total mercury (HgT) in hair was determined by CVAFS (USEPA, 2002) after digestion of the hair using the sulfuric/nitric acid digestion protocol described by Pellizzari et al. [49]. Hair samples (50–100 mg) were weighed into Teflon vials and for each sample (and reagent blank) vial, filled with acetone and allowed to soak for a minimum of 1 min. The acetone rinse was poured off and the sample allowed to dry completely before addition of the 70% HNO_3_/30% H_2_SO_4_ digestion solution. Digestion was performed at 95 °C for 3 h. After digestion, the samples were oxidized with BrCl and diluted with Type 1 water. A minimum of 16 h was allowed to pass before analysis. A small aliquot of the digest (50–100 μl) was added to a bubbler with hydroxylamine hydrochloride and ~80 ml Type 1 water. After addition of stannous chloride, the sample was purged with Ar, passing through soda lime to a single gold trap, where the mercury is collected by amalgamation with gold. The amalgam is thermally decomposed and the mercury carried by Ar to the fluorescence detector.

Methylmercury (MeHg) in hair was determined by USEPA Method 1630 (aqueous phase ethylation, followed by gas chromatographic separation and CVAFS detection) [53, 54]. The MeHg was isolated from the hair using the dilute HNO_3_ digestion method detailed in Hammerschmidt and Fitzgerald [52]. Hair samples were weighed into Teflon vials and digested with nitric acid (20%, ~3.3 M) for 8 h at 60 °C. Analysis was performed within one week of digestion. A small aliquot of the digest (50–100 μl) was added to a bubbler with 2M acetate buffer and ~80 ml Type 1 water (final pH ~4.5). Derivatization was performed with sodium tetraethylborate in KOH. The ethylated Hg species were purged with Ar and collected on a Tenax trap. The analytes were thermally desorbed from the trap and carried by Ar to the GC, where the mercury species are separated and then quantified in the fluorescence detector.

Methylmercury (MeHg) in blood was also determined by USEPA Method 1630 after digestion of the blood and isolation of the MeHg using the alkaline/alcohol (KOH/CH3OH) method described by Yu and Chandrasekhar [58]. Blood (100 μl) was delivered by pipette to polypropylene vials. Digestion was performed with 25% KOH in methanol (mass/mass) at 60 °C for 8 h. Digests were diluted with Type 1 water, and the analysis performed within one week of digestion. A small aliquot of the digest (50–100 μl) was added to a bubbler with 2 M acetate buffer and ~80 ml Type 1 water (final pH ~4.5). The analysis was completed as described above for MeHg in hair.

Both CVAAS and CVAFS are widely accepted analytical methods for quantifying total mercury concentrations in human samples such as hair. Total mercury concentrations using the two different methods were analyzed for comparability using samples from the same women (*n* = 58).

Statistics and testing were calculated and carried out using Prism ver. 8.0.1 (GraphPad, San Diego, CA, USA). Data were tested for normality (D’Agostino and Pearson test) to determine the appropriate statistical testing to perform (i.e., parametric ANOVA vs non-parametric Kruskal–Wallis). Differences in methylmercury or total mercury concentrations among the primary sites, Paramaribo, Nickerie, and the interior were examined using either ANOVA or the K–W test. When the test indicated there was a significant difference, multiple comparisons were carried out using Dunn’s correction. Correlations among methylmercury and total mercury concentrations for both hair and blood samples were conducted using either the Pearson’s test or the Spearman’s rank test. A *p* value <0.05 was used to determine statistical significance.

## Results and discussion

**Fig. 2:**
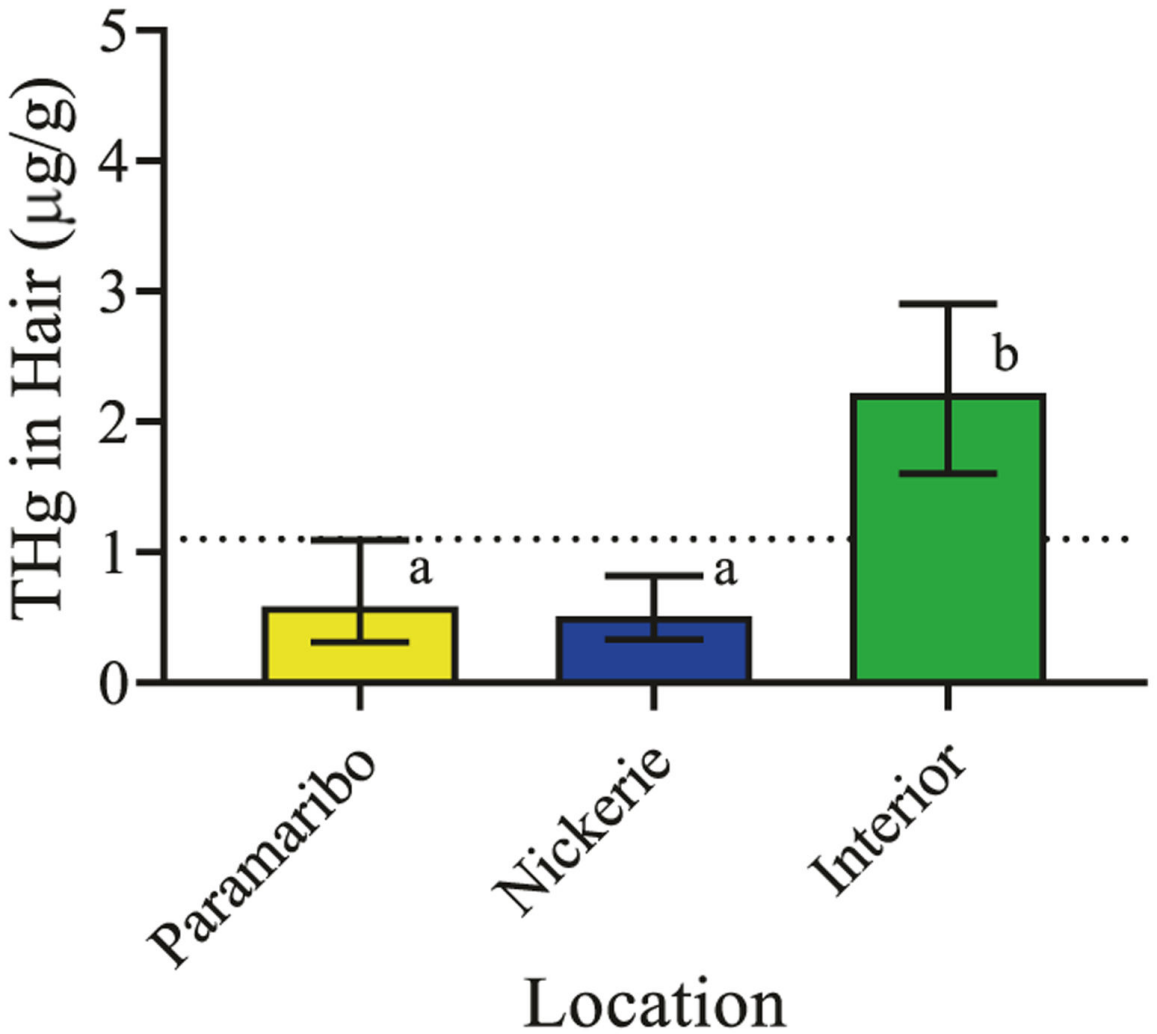
Total mercury concentrations in hair from pregnant women in the CCREOH study determined by CVAAS. Median (IQR) concentrations are presented. Dotted line represents the USEPA’s health action threshold. Different letters over bars denote significant differences at *p* < 0.0001.

Total mercury concentrations in hair determined by CVAAS did not pass normality testing (*p* < 0.0001). The Kruskal–Wallis test was used for statistical testing. Following K–W testing, multiple comparisons using Dunn’ s correction were carried out where indicated. Pregnant women in the interior have significantly higher concentrations of total mercury in hair than pregnant women from Paramaribo and Nickerie (K–W = 147.0, *p* < 0.0001, Table 1 and Fig. 2). Total mercury concentrations in hair from pregnant women from Paramaribo and Nickerie are not significantly different from one another (adjusted *p* < 0.72). Median (interquartile range) concentrations of total mercury in hair (μg/g) were 0.64 (0.36–1.09) in Paramaribo, 0.74 (0.46–1.05) in Nickerie, and 3.64 (2.08–9.24) in the interior.

**Fig. 3:**
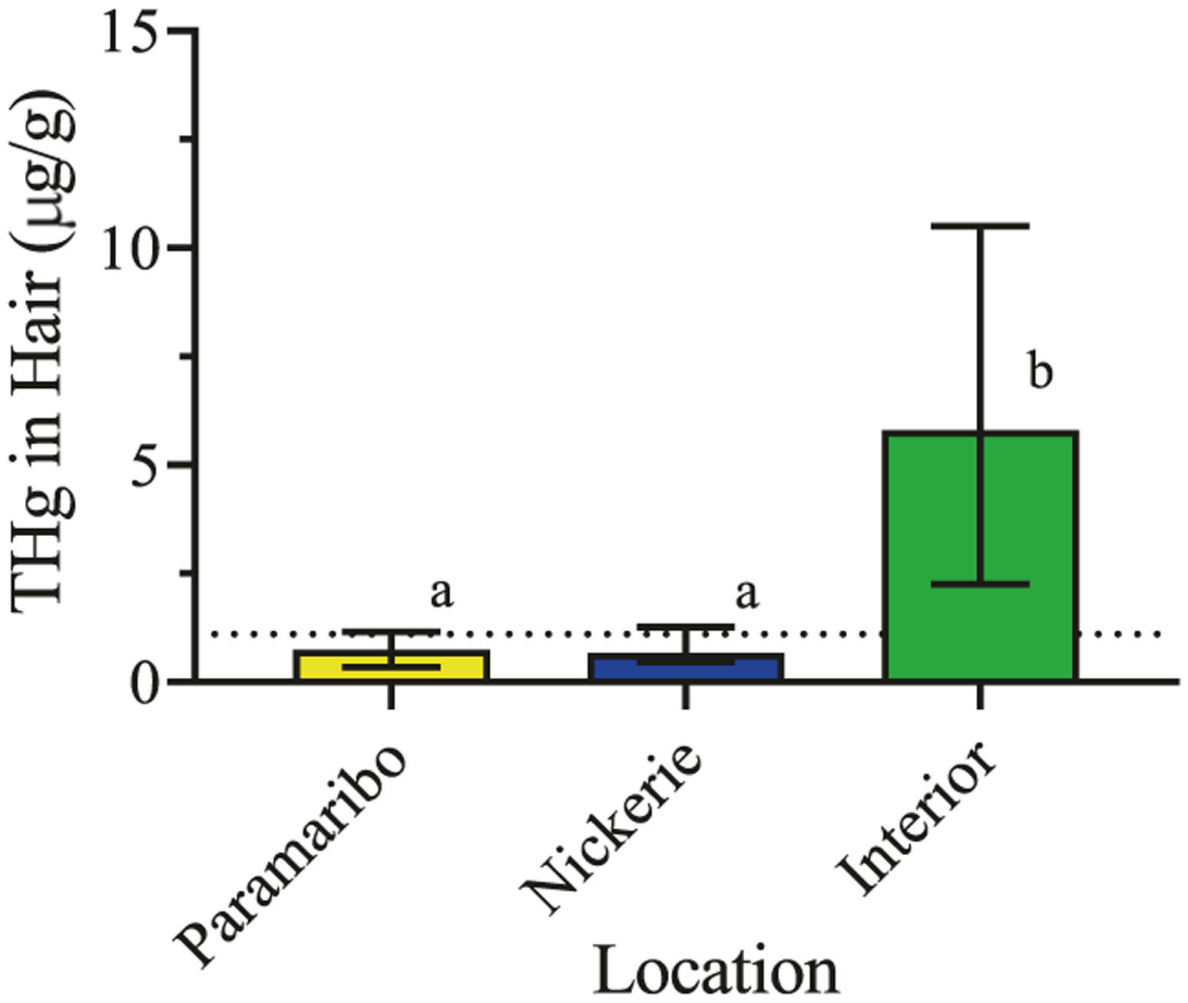
Total mercury concentrations in hair from pregnant women in the CCREOH study determined by CVAFS. Median (IQR) concentrations are presented. Dotted line represents the USEPA’s health action threshold. Different letters over bars denote significant differences at *p* < 0.0001.

Total mercury and methylmercury concentrations in hair, blood, and urine determined by CVAFS also did not pass normality testing (*p* < 0.0001). Non-parametric correlations (Spearman’s *r*) and K–W tests were used for statistical testing. Following K–W testing, multiple comparisons using Dunn’s correction were carried out where indicated. Table 2 presents the total mercury concentrations in hair, blood, and urine samples, and Table 3 presents the methylmercury concentrations in hair and blood samples. Table 4 shows the correlations among biological sample types for total mercury measurements. Total mercury concentrations in urine are modestly correlated with those in hair (*r* = 0.64, CI 0.48–0.76, *p* < 0.0001) and blood (*r* = 0.70, CI 0.56–0.80, *p* < 0.0001). Total mercury concentrations in hair and blood are highly correlated (*r* = 0.83, CI 0.73–0.89, *p* < 0.0001). Table 5 shows the correlations of total mercury and methylmercury concentrations in hair and blood samples as well as the median fraction of total mercury that is methylmercury. Total mercury and methylmercury concentrations are highly correlated in both hair (*r* = 0.97, *p* < 0.0001) and blood (*r* = 0.99, *p* < 0.0001). Methylmercury makes up most of the total mercury measured in hair and blood with median percentages (IQR) of 97.2% (74.7%–100%) and 85.5% (74.5%–98.6%), respectively. Consistent with previous work including the CVAAS results reported above, total mercury concentrations determined by CVAFS in hair from the additional 35 women in the interior are significantly higher than women in Paramaribo and Nickerie (K–W = 49.91, *p* < 0.0001, Table 2 and Fig. 3). Total mercury concentrations in hair from pregnant women from Paramaribo and Nickerie are not significantly different (adjusted *p* > 0.999). In comparing the two methods (CVAAS vs CVAFS) in a sample of 58 women, results from total mercury analyses are highly correlated and significant (*r* = 0.91, CI 0.85–0.95, *p* < 0.0001).

**Fig. 4:**
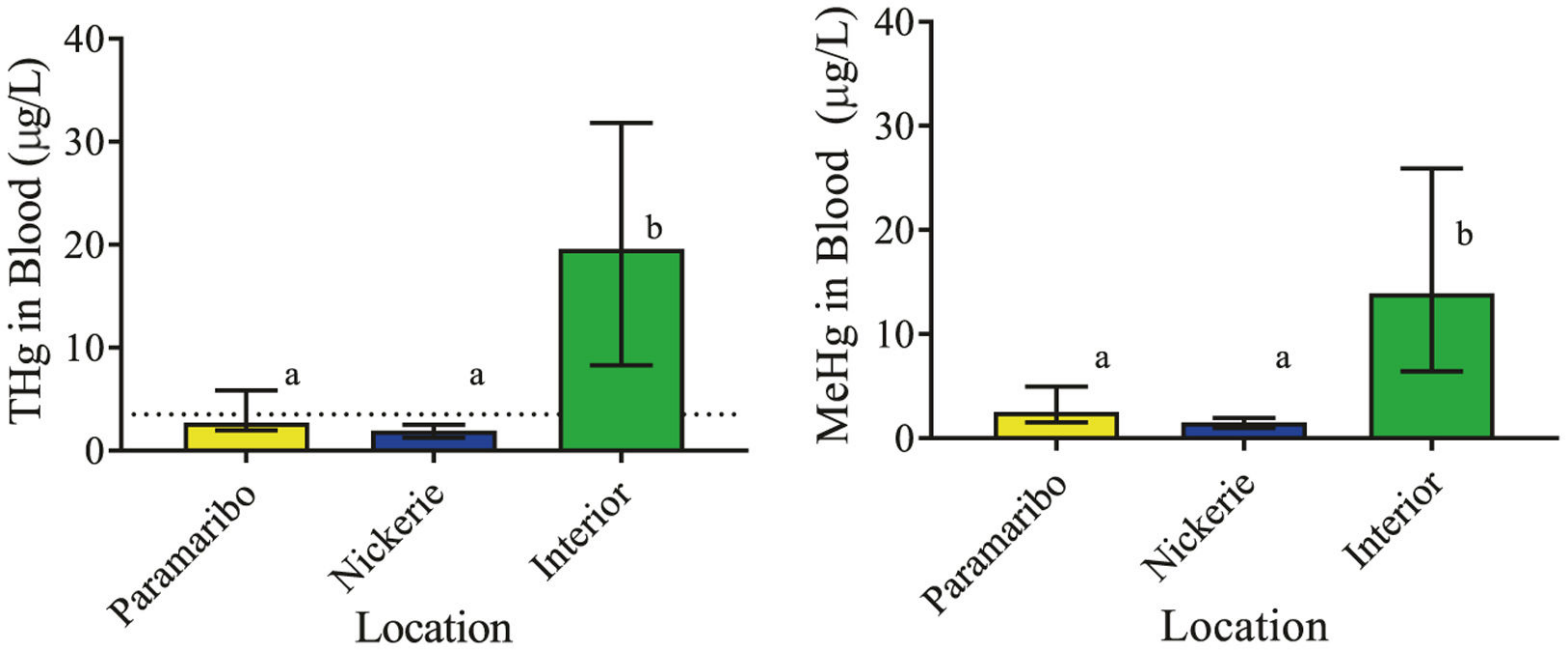
Total mercury (THg) and methylmercury (MeHg) concentrations in blood of pregnant women presented as medians (IQR). Dotted line represents the suggested health action threshold [26-28]. Different letters over bars denote significant differences at *p* < 0.0001.

Total and methylmercury concentrations in blood are also significantly higher in pregnant women from interior sites compared with women from Paramaribo and Nickerie (Table 3 and Fig. 4). Median (IQR) concentrations of total mercury in blood (μg/l) are 2.71 (1.96–5.86) in Paramaribo, 1.91 (1.26–2.51) in Nickerie, and 19.59 (8.27–31.82) in the interior (K–W = 46.79, *p* < 0.0001). Median (IQR) concentrations of methylmercury in blood (μg/l) are 2.52 (1.53–4.95) in Paramaribo, 1.50 (0.97–1.94) in Nickerie, and 13.90 (6.42–25.9) in the interior (K–W = 47.91, *p* < 0.0001). For the pregnant women in the interior, these are above health action levels established by the USEPA and the WHO [23, 24]. Total mercury concentrations in blood samples from women residing in Paramaribo and Nickerie are similar to those found in women in the US that live in coastal areas and are relatively heavy consumers of fish [7, 12, 46].

Total mercury concentrations in blood are significantly elevated in women from the interior and blood concentrations are highly correlated with total mercury concentrations in hair. Methylmercury, as opposed to elemental or inorganic mercury, is the primary form of mercury in these pregnant women making up most of the total mercury measured in blood and hair. Taken together, the total mercury and methylmercury concentrations in hair and blood suggest that exposures are mainly from dietary sources. This further supports the assertion that the majority of pregnant women in this large environmental epidemiological cohort study are primarily exposed to methylmercury from consuming fish including women in the interior where fish are contaminated by the ASGM activities in those areas [8, 43, 44]. It should be noted that pregnant women across Suriname consume similar amounts of fish, but pregnant women from the coastal regions consume mostly marine species while pregnant women from the interior communities consume almost exclusively local, freshwater species.

Other possible sources of exposure to mercury may include active participation in gold mining and/or processing, living with or in very close proximity to gold miners and/or processors, and/or the use of tainted personal care products. Based on the results of this study, these other sources would appear to be negligible or minor contributors at most. This is largely because these other sources would be expected to increase the levels of inorganic mercury in our participants’ biological samples. The strong, significant correlations between methylmercury (organic) and total mercury (organic + inorganic) in hair and blood samples in addition to the very high fractions of methylmercury that make up the total mercury measured do not support high levels of exposure to inorganic mercury in these women. Because both hair and blood have been evaluated, this suggests that exposures are ongoing and likely occur throughout most if not all of pregnancy. Blood mercury concentrations reflect current exposures (days to weeks), while hair mercury concentrations reflect exposures occurring over weeks to months. A more thorough and detailed assessment of exposure to mercury would verify this and as such is still warranted to best inform any future interventions or health advisories.

The high concentrations of total mercury and now methylmercury seen in women from the interior remain a concern. A large proportion of women from the interior have mercury concentrations which are at or above public health action levels, measured in both hair (88.6% above 1.1 μg/g ranging from 1.14 to 13.0 μg/g) and blood (97.1% above 3.5 μg/l ranging from 4.47 to 47.9 μg/l). These levels are set to protect developing children from potentially harmful exposures during gestation and pediatric development [23, 26-28]. While still debated, some previous longitudinal studies have suggested that exposure to mercury at these concentrations negatively affects neuropsychological development [11, 18, 20, 21, 59, 60]. This includes the long-running study in the Faroe Islands where concentrations of total mercury in maternal blood are similar to those found in this study [13]. Women in the Faroe Islands study, between the ages of 20 and 50 years, had a median concentration of total mercury in blood of 12.1 ug/l (range 2.6–50.1 ug/l), while women in the interior of Suriname between the ages of 16 and 45 have a median concentration of total mercury in blood of 19.6 ug/l (range 2.8–47.9 ug/l). However, dietary sources contributing to mercury exposures are quite different among these populations with women in the Faroe Islands getting a substantial amount of their mercury from consuming whale meat and those in Suriname from freshwater fish [13, 19-21, 60, 61].

Freshwater fish in the interior have high tissue concentrations of mercury likely explaining our findings that pregnant women consuming these fish have some of the highest body concentrations [8, 43, 44, 46]. Because there are often few alternative sources of protein, particularly in the interior, these findings are especially concerning. Infants and children from these mothers, and the larger cohort, are being evaluated and monitored for neurodevelopmental endpoints to improve our understanding of adverse risks to children’s neurodevelopment and the public health implications of such exposures to mercury. For some interior villages where concentrations of mercury in hair and fish are high, advisories and outreach efforts are underway.

Based on our current knowledge of mercury toxicity and fetal sensitivity, concentrations of mercury in pregnant women in the two coastal cities in Suriname, Paramaribo and Nickerie, are below those that warrant concern and are similar to heavy fish and seafood consumers in the USA. There are no ASGM operations in these areas with the exception of some gold processing shops, and women in these areas are not reliant on freshwater fish as their primary source of protein. These two areas are the largest population centers in Suriname as well. Assessing exposures to mercury in pregnant women in Suriname continues, and this is the first study to evaluate such exposures, including total and methylmercury concentrations in hair and blood, across the entire country. The effect of efforts to reduce mercury exposures through mitigating mercury use in ASGM operations and through fish consumption advisories can be objectively evaluated using applied research. Additional research is underway to more comprehensively examine beneficial and possibly neuroprotective factors found in locally harvested fish, both freshwater and marine species, that may counter the negative effects of mercury. These include essential elements such as selenium and dietary fatty acids such as ω-3 polyunsaturated fatty acids. Additional sources of mercury exposure are also being identified and evaluated (e.g., personal care products for skin lightening and whitening), which will allow for a more robust, comprehensive risk analysis and assessment. Incorporating analysis of the stable isotopes of mercury in biospecimens and environmental samples would also help in sourcing mercury inputs into the environment and better elucidate major exposure pathways. Finally, this research indicates that biomonitoring programs and studies should be developed and implemented where ASGM operations are active, especially those in the informal sector.

## Supplementary Material

Supplementary File 1

Supplementary File 2

Supplementary File 3

Supplementary File 4

## Figures and Tables

**Table 1:** Total mercury concentrations in hair samples from pregnant women in Suriname.

Location	Sample size	Median (μg/g)	IQR	Range	Non-detects
Paramaribo	525	0.63	0.36–1.09	0.00–11.88	11
Nickerie	192	0.74	0.46–1.05	0.00–14.01	1
Interior	165	3.64	2.08–9.24	0.38–31.53	0

Both the interquartile range (IQR) and total range are provided. These data include the total mercury concentrations in the sub-sample of 75 women examined for methylmercury concentrations as well.

**Table 2:** Total mercury concentrations in hair, blood, and urine samples from the subset (*n* = 75) of pregnant women in Suriname.

		Hair			Blood			Urine		
Location	Sample size	Median (μg/g)	IQR	Range	Median (μg/l)	IQR	Range	Median (μg/l)	IQR	Range
Paramaribo	20	0.75	0.34–1.16	0.04–1.56	2.71	1.96–5.86	0.51–19.92	1.28	0.71–2.75	0.18–4.37
Nickerie	20	0.66	0.46–1.27	0.05–2.54	1.91	1.26–2.51	0.80–7.20	1.48	0.52–2.00	0.11–7.80
Interior	35	5.81	2.26–10.50	1.25–18.2	19.60	8.27–47.90	2.80–47.90	5.31	2.52–16.11	0.27–66.67

Both the interquartile range (IQR) and total range are provided.

**Table 3:** Methylmercury concentrations in hair and blood samples from the subset (*n* = 75) of pregnant women in Suriname.

		Hair			Blood		
Location	Sample size	Median (μg/g)	IQR	Range	Median (μg/l)	IQR	Range
Paramaribo	20	0.58	0.17–0.91	0.05–1.43	2.52	1.53–4.95	0.59–12.30
Nickerie	20	0.74	0.35–1.11	0.06–2.62	1.50	0.97–6.57	0.58–6.57
Interior	35	5.53	2.01–12.15	0.51–21.46	13.90	6.42–25.90	2.51–49.50

Both the interquartile range (IQR) and total range are provided.

**Table 4:** Correlation matrix (Spearman’s *r*) for biological sample types and total mercury concentrations.

Sample type	Urine	Hair
Hair	0.643 (0.482–0.762)	
Blood	0.703 (0.562–0.805)	0.825 (0.733–0.888)

Confidence intervals (95%) are provided. Correlations are significant at *p* < 0.0001.

**Table 5:** Correlation matrix (Spearman’s *r*) for total mercury and methylmercury concentrations in hair and blood.

Methylmercury	Total mercury (*r*)	% of total mercury
Hair	0.974 (0.959–0.984)	97.2 (23.3–100)
Blood	0.986 (0.978–0.991)	85.5 (47.3–100)

Confidence intervals (95%) are provided. Median methylmercury percentages, (total range) of total mercury concentrations are shown. Correlations are significant at *p* < 0.0001.

## References

[1] Clarkson TW. The three modern faces of mercury. Environ Health Perspect. 2002;110(Suppl 1):11–23. 11834460 10.1289/ehp.02110s111 PMC1241144

[2] Clarkson TW, Magos L. The toxicology of mercury and its chemical compounds. Crit Rev Toxicol. 2006;36:609–62. 16973445 10.1080/10408440600845619

[3] Bakir F, Damluji SF, Amin-Zaki L, Murtadha M, Khalidi A, Al-Rawi NY, Methylmercury poisoning in Iraq. Science. 1973;181:230–41. 4719063 10.1126/science.181.4096.230

[4] Castaño A, Cutanda F, Esteban M, Pärt P, Navarro C, Gómez S, Fish consumption patterns and hair mercury levels in children and their mothers in 17 EU countries. Environ Res. 2015;141:58–68. 25667172 10.1016/j.envres.2014.10.029

[5] Harada M Minamata disease: methylmercury poisoning in Japan caused by environmental pollution. Crit Rev Toxicol. 1995;25:1–24. 7734058 10.3109/10408449509089885

[6] Hawkins WB. Global environmental public health issues related to gold mining and mercury contamination in Indigenous communities in Suriname, South America School of Public Health and Tropical Medicine. New Orleans, LA: Tulane University; 2012.

[7] Mahaffey KR, Clickner RP, Jeffries RA. Adult women’s blood mercury concentrations vary regionally in the United States: association with patterns of fish consumption (NHANES 1999-2004). Environ Health Perspect. 2008;117:47–53. 19165386 10.1289/ehp.11674 PMC2627864

[8] Ouboter PE, Landburg GA. Mercury poisoning: a threat to Brownsweg villagers. Paramaribo, Suriname: World Wildlife Foundation; 2010.

[9] Ricketts P, Basu N, Fletcher H, Voutchkov M, Bassaw B. Assessment of fish consumption and mercury exposure among pregnant women in Jamaica and Trinidad and Tobago. Chemosphere. 2016;164:462–8. 27612166 10.1016/j.chemosphere.2016.08.054

[10] Strain J, Yeates AJ, van Wijngaarden E, Thurston SW, Mulhern MS, McSorley EM, Prenatal exposure to methyl mercury from fish consumption and polyunsaturated fatty acids: associations with child development at 20 mo of age in an observational study in the Republic of Seychelles. Am J Clin Nutr. 2015;101:530–7. 25733638 10.3945/ajcn.114.100503 PMC4340059

[11] vvan Wijngaarden E, Thurston SW, Myers GJ, Harrington D, Cory-Slechta DA, Strain JJ, Methyl mercury exposure and neurodevelopmental outcomes in the Seychelles Child Development Study Main cohort at age 22 and 24 years. Neurotoxicology Teratol. 2017;59:35–42. 10.1016/j.ntt.2016.10.011 PMC5235948 27989696

[12] Zilversmit L, Wickliffe J, Shankar A, Taylor R, Harville E. Correlations of biomarkers and self-reported seafood consumption among pregnant and non-pregnant women in southeastern Louisiana after the Gulf oil spill: the GROWH study. Int J Environ Res Public Health. 2017;14:784. 10.3390/ijerph14070784 PMC5551222 28708119

[13] Grandjean P, Weihe P, Jorgensen PJ, Clarkson T, Cernichiari E, Videro T. Impact of maternal seafood diet on fetal exposure to mercury, selenium, and lead. Arch Environ Health. 1992;47:185–95. 1596101 10.1080/00039896.1992.9938348

[14] Dong Z, Jim RC, Hatley EL, Backus ASN, Shine JP, Spengler JD, A longitudinal study of mercury exposure associated with consumption of freshwater fish from a reservoir in rural south central USA. Environ Res. 2015;136:155–62. 25460632 10.1016/j.envres.2014.09.029 PMC4348364

[15] UNEP. Global mercury assessment 2018. Geneva: UN Enviroment Program, Chemicals and Health Branch; 2019.

[16] Lawley R, Curtis L, Davis J. The food safety hazard guidebook. Cambridge: The Royal Society of Chemistry; 2012 533 p.

[17] Ratcliffe HE, Swanson GM, Fischer LJ. Human exposure to mercury: a critical assessment of the evidence of adverse health effects. J Toxicol Environ Health. 1996;49:221–70. 8876653 10.1080/713851079

[18] Crump KS, Kjellström T, Shipp AM, Silvers A, Stewart A. Influence of prenatal mercury exposure upon scholastic and psychological test performance: benchmark analysis of a New Zealand cohort. Risk Anal. 1998;18:701–13. 9972579 10.1023/b:rian.0000005917.52151.e6

[19] Grandjean P, Weihe P, Debes F, Choi AL, Budtz-Jørgensen E. Neurotoxicity from prenatal and postnatal exposure to methylmercury. Neurotoxicology Teratol. 2014;43:39–44. 10.1016/j.ntt.2014.03.004 PMC4066386 24681285

[20] Grandjean P, Weihe P, White RF, Debes F. Cognitive performance of children prenatally exposed to “safe” levels of methylmercury. Environ Res. 1998;77:165–72. 9600810 10.1006/enrs.1997.3804

[21] Grandjean P, Weihe P, White RF, Debes F, Araki S, Yokoyama K, Cognitive deficit in 7-year-old children with prenatal exposure to methylmercury. Neurotoxicology Teratol. 1997;19:417–28. 10.1016/s0892-0362(97)00097-4 9392777

[22] Marsh DO, Clarkson TW, Cox C, Myers GJ, Amin-Zaki L, Al-Tikriti S. Fetal methylmercury poisoning: relationship between concentration in single strands of maternal hair and child effects. Arch Neurol. 1987;44:1017–22. 2443112 10.1001/archneur.1987.00520220023010

[23] USEPA. Mercury study report to congress volume v: health effects of mercury and mercury compounds. EPA-452/R-97–007 ed. Washington, D.C.: USEPA; 1997 p. 1–348.

[24] WHO. Evaluation of certain food additives and contaminants: sixty-seventh report of the Joint FAO/WHO Expert Committee on Food Additives. 2006.

[25] USEPA. Resources for mercury science and research. USEPA; 2019 https://www.epa.gov/mercury/resources-mercury-science-and-research.

[26] Donohue A, Wagner CL, Burch JB, Rothenberg SE. Blood total mercury and methylmercury among pregnant mothers in Charleston, South Carolina, USA. J Expo Sci Environ Epidemiol. 2018;28:494–504. 29670220 10.1038/s41370-018-0033-1 PMC7425126

[27] Mortensen ME, Caudill SP, Caldwell KL, Ward CD, Jones RL. Total and methyl mercury in whole blood measured for the first time in the U.S. population: NHANES 2011–2012. Environ Res. 2014;134:257–64. 25173092 10.1016/j.envres.2014.07.019 PMC5584810

[28] Mahaffey KR, Clickner RP, Bodurow CC. Blood organic mercury and dietary mercury intake: National Health and Nutrition Examination Survey, 1999 and 2000. Environ health Perspect. 2004;112:562–70. 15064162 10.1289/ehp.6587 PMC1241922

[29] UNEP. Global mercury assessment: sources, emissions, releases and environmental transport. Geneva: UN Environment Program, Chemicals Branch; 2013.

[30] Manceau A, Enescu M, Simionovici A, Lanson M, Gonzalez-Rey M, Rovezzi M, Chemical forms of mercury in human hair reveal sources of exposure. Environ Sci Technol. 2016;50:10721–9. 27676331 10.1021/acs.est.6b03468

[31] Laffont L, Sonke JE, Maurice L, Monrroy SL, Chincheros J, Amouroux D, Hg speciation and stable isotope signatures in human hair as a tracer for dietary and occupational exposure to mercury. Environ Sci Technol. 2011;45:9910–6. 22003970 10.1021/es202353m

[32] Airey D Mercury in human hair due to environment and diet: a review. Environ Health Perspect. 1983;52:303–16. 6653535 10.1289/ehp.8352303 PMC1569325

[33] Fitzgerald WF, Clarkson TW. Mercury and monomethylmercury: present and future concerns. Environ Health Perspect. 1991;96: 159–66. 1820259 10.1289/ehp.9196159 PMC1568233

[34] Cordy P, Veiga MM, Salih I, Al-Saadi S, Console S, Garcia O, Mercury contamination from artisanal gold mining in Antioquia, Colombia: the world’s highest per capita mercury pollution. Sci Total Environ. 2011;410–411:154–60. 22000915 10.1016/j.scitotenv.2011.09.006

[35] Ha E, Basu N, Bose-O’Reilly S, Dórea JG, McSorley E, Sakamoto M, Current progress on understanding the impact of mercury on human health. Environ Res. 2017;152:419–33. 27444821 10.1016/j.envres.2016.06.042

[36] Seccatore J, Veiga M, Origliasso C, Marin T, De Tomi G. An estimation of the artisanal small-scale production of gold in the world. Sci Total Environ. 2014;496:662–7. 24867677 10.1016/j.scitotenv.2014.05.003

[37] Spiegel S, Keane S, Metcalf S, Veiga M, Yassi A. The Minamata convention on mercury: time to seek solutions with artisanal mining communities. Environ Health Perspect. 2014;122:A203–A4. 25229087 10.1289/ehp.1408514 PMC4123035

[38] Steckling N, Boese-O’Reilly S, Gradel C, Gutschmidt K, Shinee E, Altangerel E, Mercury exposure in female artisanal small-scale gold miners (ASGM) in Mongolia: an analysis of human biomonitoring (HBM) data from 2008. Sci Total Environ. 2011; 409:994–1000. 21183207 10.1016/j.scitotenv.2010.11.029

[39] Steckling N, Tobollik M, Plass D, Hornberg C, Ericson B, Fuller R, Global burden of disease of mercury used in artisanal small-scale gold mining. Ann Glob Health. 2017;83:234–47. 28619398 10.1016/j.aogh.2016.12.005

[40] Veiga MM, Angeloci-Santos G, Meech JA. Review of barriers to reduce mercury use in artisanal gold mining. Extractive Industries Soc. 2014;1:351–61.

[41] Gibb H, O’Leary KG. Mercury exposure and health impacts among individuals in the artisanal and small-scale gold mining community: a comprehensive review. Environ Health Perspect. 2014;122:667–72. 24682486 10.1289/ehp.1307864 PMC4080518

[42] Legg ED, Ouboter PE, Wright MAP. Small-scale gold mining related mercury contamination in the Guianas: a review. Paramaribo, Suriname: World Wildlife Fund; 2015.

[43] Ouboter PE, Landburg GA, Quik JHM, Mol JHA, van der Lugt F. Mercury levels in pristine and gold mining impacted aquatic ecosystems of Suriname, South America. Ambio. 2012;41:873–82. 22669686 10.1007/s13280-012-0299-9 PMC3492555

[44] Ouboter PE, Landburg GA, White C, Mol J, van der Lugt F, Quik JHM. Final technical report mercury pollution in the greenstone belt. Paramaribo, Suriname: World Wildlife Fund; 2007.

[45] Mohan S, Tiller M, Van der Voet G, Kanhai H. Mercury exposure of mothers and newborns in Surinam: a pilot study. Clin Toxicol. 2005;43:101–4. 15822761

[46] Ouboter P, Landburg G, Satnarain G, Starke S, Nanden I, Simon-Friedt B, Mercury levels in women and children from interior villages in Suriname, South America. Int J Environ Res Public Health. 2018;15:1007. 10.3390/ijerph15051007 PMC5982046 29772808

[47] Basu N, Horvat M, Evers DC, Zastenskaya I, Weihe P, Tempowski J. A state-of-the-science review of mercury biomarkers in human populations worldwide between 2000 and 2018. Environ Health Perspect. 2018;126:106001. 30407086 10.1289/EHP3904 PMC6371716

[48] Lichtveld MY, Zijlmans CWR, Ouboter PE, Hawkins WB, Wickliffe JK, Abdoel Wahid F, The Caribbean consortium for research in environmental and occupational health: examining the impact of neurotoxicant exposures on maternal and child health in Suriname NIEHS Environmental Health Science FEST; Durham, NC: NIEHS; 2016.

[49] Pellizzari ED, Fernando R, Cramer GM, Meaburn GM, Bangerter K. Analysis of mercury in hair of EPA Region V population. J Expo Sci Environ Epidemiol. 1999;9:393–401. 10.1038/sj.jea.7500056 10554142

[50] Kim B-G, Jo E-M, Kim G-Y, Kim D-S, Kim Y-M, Kim R-B, Analysis of methylmercury concentration in the blood of Koreans by using cold vapor atomic fluorescence spectrophotometry. Ann Lab Med. 2012;32:31–7. 22259776 10.3343/alm.2012.32.1.31 PMC3255500

[51] Liang L, Evens C, Lazoff S, Woods JS, Cernichiari E, Horvat M, Determination of methyl mercury in whole blood by ethylation-GC-CVAFS after alkaline digestion-solvent extraction. J Anal Toxicol. 2000;24:328–32. 10926355 10.1093/jat/24.5.328

[52] Hammerschmidt CR, Fitzgerald WF. Bioaccumulation and trophic transfer of methylmercury in Long Island Sound. Arch Environ Contamination Toxicol. 2006;51:416–24. 10.1007/s00244-005-0265-7 16823518

[53] USEPA. Method 1630, methyl mercury in water by distillation, aqueous ethylation, purge and trap, and CVAFS. EPA-821-R-01-020 ed. Washington, D.C.: U.S. Environmental Protection Agency, Office of Water; 2001.

[54] USGS. Determination of methyl mercury by aqueous phase ethylation, followed by gas chromatographic separation with cold vapor atomic fluorescence detection. Open-File Report 01-445 ed. Reston, VA: U.S. Department of the Interior; 2002.

[55] USEPA. Method 1631, Revision E: mercury in water by oxidation, purge and trap, and cold vapor atomic fluorescence spectrometry. EPA-821-R-02-019 ed. Washington, D.C.: U.S. Environmental Protection Agency, Office of Water; 2002.

[56] CDC. Laboratory procedure manual, cadmium, lead, manganese, mercury, and selenium in whole blood Blood metals panel 3. DLS 3016.8-04 ed. Atlanta, GA: National Center for Environmental Health, Centers for Disease Control; 2014.

[57] CDC. Laboratory procedure manual, iodine and mercury in urine (MEC and 24-Hour) Iodine and mercury in urine by ICP-DRC-MS. DLS 3002.7-02 ed. Atlanta, GA: National Center for Environmental Health, Centers for Disease Control; 2014.

[58] Yu X, Chandrasekhar TM. Analysis of methyl mercury in sediment and tissue by KOH/CH3OH digestion followed by aqueous phase ethylation. Tallahassee, FL: Florida Department of Environmental Protection, Bureau of Laboratories; 2005.

[59] Davidson PW, Strain JJ, Myers GJ, Thurston SW, Bonham MP, Shamlaye CF, Neurodevelopmental effects of maternal nutritional status and exposure to methylmercury from eating fish during pregnancy. NeuroToxicology. 2008;29:767–75. 18590763 10.1016/j.neuro.2008.06.001 PMC2580738

[60] Grandjean P, Weihe P, Nielsen F, Heinzow B, Debes F, Budtz-Jorgensen E. Neurobehavioral deficits at age 7 years associated with prenatal exposure to toxicants from maternal seafood diet. Neurotoxicology Teratol. 2012;34:466–72. 10.1016/j.ntt.2012.06.001 PMC3407364 22705177

[61] Grandjean P, Jørgensen PJ, Weihe P. Human milk as a source of methylmercury exposure in infants. Environ Health Perspect. 1994;102:74–7. 9719671 10.1289/ehp.9410274 PMC1567218

