# Supplementary material for: Exposure to total and methylmercury among pregnant women in Suriname: sources and public health implications
Source: J Expo Sci Environ Epidemiol. Author manuscript; Available in PMC 2021 Feb 1. (PMC7704553; doi:10.1038/s41370-020-0233-3)
Supplement: Supplementary File 1 [file NIHMS1599501-supplement-Supplementary_File_1.docx]

| Table S1. Evaluation of exogenous total mercury in a subset of hair samples with varying concentrations of total mercury using the standard acetone rinse method in comparison to a more rigorous acetone and detergent rinse method. Hair samples were soaked in acetone (5 ml for 5 min) and either collected and processed for analysis or subjected to a second wash in 5 ml of 0.5% Triton X and sonicated for 5 min. Samples were rinsed in 10 ml milliQwater (5 ml twice). Samples were then dry digested then diluted to 15 ml with milliQwater and analyzed. Very little mercury was found in any of the rinses (on average ~0.4%) thus there appears to be very little exogenous mercury in these hair samples. It should be noted that these are hair samples from participants in the interior region of Suriname including 3 samples from participants in areas where there is active ASGM activity. | | | | | |
| --- | --- | --- | --- | --- | --- |
|  | New Analyses (09/07/2019) | | Original Analyses (09/21/2018) | Acetone Rinse HgT (percentage of rinsed hair) | Hair HgT (Acetone+Detergent)/Acetone (RPD) |
|  | Hair HgT (µg/g) after acetone & detergent rinses | Acetone Rinse µg/g | Hair HgT (µg/g) after acetone rinse |  |  |
| Sample ID |  |  |  |  |  |
| I6-2077-1 | 4.86 | 0.0196 | 4.10 | 0.40 | 16.9 |
| I6-2096-1 | 7.00 | 0.0126 | 6.43 | 0.18 | 8.5 |
| I6-2116-1 | 12.84 | 0.0275 | 12.20 | 0.21 | 5.1 |
| I6-2116-1 DUP | 13.09 | 0.0342 | 12.20 | 0.26 | 7.1 |
| I6-2117-1 | 3.02 | 0.0162 | 2.55 | 0.54 | 16.7 |
| I6-2122-1 | 12.98 | 0.0370 | 12.80 | 0.28 | 1.4 |
| I6-2124-1 | 20.07 | 0.0713 | 18.20 | 0.36 | 9.7 |
| I6-2130-1 | 11.66 | 0.0783 | 11.20 | 0.67 | 4.0 |
| I6-2130-1 DUP | 11.51 | 0.0533 | 11.20 | 0.46 | 2.7 |
| I6-2132-1 | 5.13 | 0.0361 | 4.65 | 0.70 | 9.8 |
