# Supplementary material for: Exposure to total and methylmercury among pregnant women in Suriname: sources and public health implications
Source: J Expo Sci Environ Epidemiol. Author manuscript; Available in PMC 2021 Feb 1. (PMC7704553; doi:10.1038/s41370-020-0233-3)
Supplement: Supplementary File 2 [file NIHMS1599501-supplement-Supplementary_File_2.docx]

| Table S2. Quality assurance and quality control indicators for all analyses of methylmercury in hair samples from research participants in Suriname. All QA/QC indicators are met regarding the laboratory methods used in this study. | | | | | | | | | | |
| --- | --- | --- | --- | --- | --- | --- | --- | --- | --- | --- |
| Type | Name/ID | Final Result | Units | Spike Concentration | Source Result | % REC | % REC Limit | RPD | RPD Limit | Analysis Date |
| Lab Fortified Blank | LFB | 1008.189 | µg/Kg | 1000 |  | 100.8189 | 67-133 |  |  | 11/16/2018 |
| Lab Reagent Blank | LRB1 | 0.274 | pg |  |  |  |  |  |  | 11/16/2018 |
| Lab Reagent Blank | LRB2 | 0.267 | pg |  |  |  |  |  |  | 11/16/2018 |
| Matrix Spike | I621211 | 2208.457 | µg/Kg | 1113 | 334.5327 | 78.51974 | 65-135 |  |  | 11/16/2018 |
| Method Duplicate | I620771 | 3008.043 | µg/Kg |  | 3538.436 |  |  | 16.2 | 35 | 11/16/2018 |
| Ongoing Precision and Recovery | OPR1 | 31 | µg/Kg | 30 |  | 103.4361 | 67-133 |  |  | 11/16/2018 |
| Ongoing Precision and Recovery | OPR2 | 31.2 | µg/Kg | 30 |  | 103.9414 | 67-133 |  |  | 11/16/2018 |
| Ongoing Precision and Recovery | OPR3 | 29.5 | µg/Kg | 30 |  | 98.48415 | 67-133 |  |  | 11/16/2018 |
| Standard Reference Material | DORM-3 | 356.5303 | µg/Kg | 355 |  | 100.4311 | 67-133 |  |  | 11/16/2018 |
| Standard Reference Material | BCR397 | 882.2291 | µg/Kg | 870 |  | 101.4056 | 67-133 |  |  | 11/16/2018 |
| Lab Fortified Blank | LFB | 1320.47 | µg/Kg | 1000 |  | 132.047 | 67-133 |  |  | 11/27/2018 |
| Lab Reagent Blank | LRB1 | 0 | pg |  |  |  |  |  |  | 11/27/2018 |
| Lab Reagent Blank | LRB2 | 3.2 | pg |  |  |  |  |  |  | 11/27/2018 |
| Matrix Spike | N511321 | 181.1524 | µg/Kg | 1042 | 138.8324 | 100.0307 | 65-135 |  |  | 11/27/2018 |
| Matrix Spike | R732171 | 456.421 | µg/Kg | 1689 | 430.3176 | 119.9588 | 65-135 |  |  | 11/27/2018 |
| Method Duplicate | I620541 | 1148.351 | µg/Kg |  | 1208.162 |  |  | 5.08 | 35 | 11/27/2018 |
| Method Duplicate | N511521 | 296.1761 | µg/Kg |  | 256.3151 |  |  | 14.4 | 35 | 11/27/2018 |
| Ongoing Precision and Recovery | OPR1 | 33.5 | µg/Kg | 30 |  | 111.5326 | 67-133 |  |  | 11/27/2018 |
| Ongoing Precision and Recovery | OPR2 | 26.8 | µg/Kg | 30 |  | 89.21124 | 67-133 |  |  | 11/27/2018 |
| Ongoing Precision and Recovery | OPR3 | 29.2 | µg/Kg | 30 |  | 97.21113 | 67-133 |  |  | 11/27/2018 |
| Standard Reference Material | BCR397 | 989.1468 | µg/Kg | 870 |  | 113.695 | 67-133 |  |  | 11/27/2018 |
| Standard Reference Material | DORM-3 | 394.5907 | µg/Kg | 355 |  | 111.1523 | 67-133 |  |  | 11/27/2018 |
| Lab Fortified Blank | LFB | 1025.095 | µg/Kg | 1000 |  | 102.5095 | 67-133 |  |  | 12/4/2018 |
| Lab Reagent Blank | LRB1 | 0.211 | pg |  |  |  |  |  |  | 12/4/2018 |
| Lab Reagent Blank | LRB2 | 0.167 | pg |  |  |  |  |  |  | 12/4/2018 |
| Matrix Spike | R732421 | 3391.804 | µg/Kg | 2304 | 1259.421 | 92.55135 | 65-135 |  |  | 12/4/2018 |
| Method Duplicate | I621201 | 6396.114 | µg/Kg |  | 5720.54 |  |  | 11.2 | 35 | 12/4/2018 |
| Ongoing Precision and Recovery | OPR1 | 33.4 | µg/Kg | 30 |  | 111.2756 | 67-133 |  |  | 12/4/2018 |
| Ongoing Precision and Recovery | OPR2 | 31 | µg/Kg | 30 |  | 103.2458 | 67-133 |  |  | 12/4/2018 |
| Standard Reference Material | BCR397 | 681.2228 | µg/Kg | 870 |  | 78.30147 | 67-133 |  |  | 12/4/2018 |
| Standard Reference Material | DORM-3 | 310.3531 | µg/Kg | 355 |  | 87.42341 | 67-133 |  |  | 12/4/2018 |
| Lab Fortified Blank | LFB | 966.2744 | µg/Kg | 1000 |  | 96.62744 | 67-133 |  |  | 12/6/2018 |
| Lab Reagent Blank | LRB1 | 0.264 | pg |  |  |  |  |  |  | 12/6/2018 |
| Lab Reagent Blank | LRB2 | 0 | pg |  |  |  |  |  |  | 12/6/2018 |
| Matrix Spike | I620621 | 6981.808 | µg/Kg | 2212 | 4606.557 | 107.3803 | 65-135 |  |  | 12/6/2018 |
| Method Duplicate | R732161 | 695.6301 | µg/Kg |  | 488.5998 |  |  | 35 | 35 | 12/6/2018 |
| Ongoing Precision and Recovery | OPR1 | 32.3 | µg/Kg | 30 |  | 107.6086 | 67-133 |  |  | 12/6/2018 |
| Ongoing Precision and Recovery | OPR2 | 29.1 | µg/Kg | 30 |  | 97.0996 | 67-133 |  |  | 12/6/2018 |
| Standard Reference Material | BCR397 | 771.3653 | µg/Kg | 870 |  | 88.66267 | 67-133 |  |  | 12/6/2018 |
| Standard Reference Material | DORM-3 | 299.2527 | µg/Kg | 355 |  | 84.29654 | 67-133 |  |  | 12/6/2018 |
| Lab Fortified Blank | LFB | 1053.601 | µg/Kg | 1000 |  | 105.3601 | 67-133 |  |  | 12/10/2018 |
| Lab Reagent Blank | LRB1 | 0.00442 | pg |  |  |  |  |  |  | 12/10/2018 |
| Lab Reagent Blank | LRB2 | 0.212 | pg |  |  |  |  |  |  | 12/10/2018 |
| Matrix Spike | I621161 | 18928.34 | µg/Kg | 3788 | 15889.4 | 80.22552 | 65-135 |  |  | 12/10/2018 |
| Method Duplicate | R740441 | 287.0627 | µg/Kg |  | 232.4755 |  |  | 21 | 35 | 12/10/2018 |
| Ongoing Precision and Recovery | OPR1 | 35 | µg/Kg | 30 |  | 116.8026 | 67-133 |  |  | 12/10/2018 |
| Ongoing Precision and Recovery | OPR2 | 32.2 | µg/Kg | 30 |  | 107.4834 | 67-133 |  |  | 12/10/2018 |
| Standard Reference Material | BCR397 | 723.7867 | µg/Kg | 870 |  | 83.19388 | 67-133 |  |  | 12/10/2018 |
| Standard Reference Material | DORM-3 | 306.899 | µg/Kg | 355 |  | 86.45042 | 67-133 |  |  | 12/10/2018 |
| Lab Fortified Blank | LFB | 1068.597 | µg/Kg | 1000 |  | 106.8597 | 67-133 |  |  | 12/12/2018 |
| Lab Reagent Blank | LRB1 | 0.00263 | pg |  |  |  |  |  |  | 12/12/2018 |
| Lab Reagent Blank | LRB2 | 0.000163 | pg |  |  |  |  |  |  | 12/12/2018 |
| Matrix Spike | I621321 | 7538.434 | µg/Kg | 2212 | 5462.705 | 93.83945 | 65-135 |  |  | 12/12/2018 |
| Method Duplicate | I621031 | 12214.5 | µg/Kg |  | 14671.97 |  |  | 18.3 | 35 | 12/12/2018 |
| Ongoing Precision and Recovery | OPR1 | 32.8 | µg/Kg | 30 |  | 109.4089 | 67-133 |  |  | 12/12/2018 |
| Ongoing Precision and Recovery | OPR2 | 32.5 | µg/Kg | 30 |  | 108.459 | 67-133 |  |  | 12/12/2018 |
| Standard Reference Material | BCR397 | 683.5032 | µg/Kg | 870 |  | 78.56359 | 67-133 |  |  | 12/12/2018 |
| Standard Reference Material | DORM-3 | 269.5698 | µg/Kg | 355 |  | 75.93515 | 67-133 |  |  | 12/12/2018 |
| Lab Fortified Blank | LFB | 1136.079 | µg/Kg | 1000 |  | 113.6079 | 67-133 |  |  | 12/18/2018 |
| Lab Reagent Blank | LRB1 | 0.00166 | pg |  |  |  |  |  |  | 12/18/2018 |
| Lab Reagent Blank | LRB2 | 0.0214 | pg |  |  |  |  |  |  | 12/18/2018 |
| Matrix Spike | R732831 | 1395.029 | µg/Kg | 595 | 634.3002 | 127.8536 | 65-135 |  |  | 12/18/2018 |
| Method Duplicate | P101671 | 1240.645 | µg/Kg |  | 1021.27 |  |  | 19.4 | 35 | 12/18/2018 |
| Ongoing Precision and Recovery | OPR1 | 33.4 | µg/Kg | 30 |  | 111.217 | 67-133 |  |  | 12/18/2018 |
| Ongoing Precision and Recovery | OPR2 | 29.2 | µg/Kg | 30 |  | 97.22394 | 67-133 |  |  | 12/18/2018 |
| Standard Reference Material | BCR397 | 660.5915 | µg/Kg | 870 |  | 75.93005 | 67-133 |  |  | 12/18/2018 |
| Standard Reference Material | DORM-3 | 310.0884 | µg/Kg | 355 |  | 87.34883 | 67-133 |  |  | 12/18/2018 |
