# Supplementary material for: Exposure to total and methylmercury among pregnant women in Suriname: sources and public health implications
Source: J Expo Sci Environ Epidemiol. Author manuscript; Available in PMC 2021 Feb 1. (PMC7704553; doi:10.1038/s41370-020-0233-3)
Supplement: Supplementary File 3 [file NIHMS1599501-supplement-Supplementary_File_3.docx]

| Table S3. Quality assurance and quality control indicators for all analyses of methylmercury in blood samples from research participants in Suriname. All QA/QC indicators are met regarding the laboratory methods used in this study. | | | | | | | | | | |
| --- | --- | --- | --- | --- | --- | --- | --- | --- | --- | --- |
| Type | Name/ID | Final Result | Units | Spike Concentration | Source Result | % REC | % REC Limit | RPD | RPD Limit | Analysis Date |
| Lab Fortified Blank | LFB1 | 3,744.43 | ng/L | 5000 |  | 74.88862 | 67-133 |  |  | 10/3/2018 |
| Lab Reagent Blank | LRB1 | 0.0548 | pg |  |  |  |  |  |  | 10/3/2018 |
| Lab Reagent Blank | LRB2 | 0.00883 | pg |  |  |  |  |  |  | 10/3/2018 |
| Matrix Spike | R732291 | 6,510.05 | ng/L | 5000 | 1,324.42 | 103.7125 | 71-125 |  |  | 10/3/2018 |
| Matrix Spike | R732301 | 9,171.10 | ng/L | 5000 | 3,755.96 | 108.3028 | 71-125 |  |  | 10/3/2018 |
| Method Duplicate | R731631 | 1,975.80 | ng/L |  | 1,728.60 |  |  | 13.3 | 35 | 10/3/2018 |
| Method Duplicate | R732831 | 2,696.46 | ng/L |  | 2,584.18 |  |  | 4.25 | 35 | 10/3/2018 |
| Ongoing Precision and Recovery | OPR1 | 30.1 | ng/L | 30 |  | 100.2493 | 67-133 |  |  | 10/3/2018 |
| Ongoing Precision and Recovery | OPR2 | 8.46 | ng/L | 10 |  | 84.64394 | 67-133 |  |  | 10/3/2018 |
| Ongoing Precision and Recovery | OPR3 | 7.18 | ng/L | 10 |  | 71.79967 | 67-133 |  |  | 10/3/2018 |
| Standard Reference Material | NIST955c 1 | 4,614.07 | ng/L | 4500 |  | 102.5348 | 67-133 |  |  | 10/3/2018 |
| Lab Fortified Blank | LFB1 | 4,106.36 | ng/L | 5000 |  | 82.12719 | 67-133 |  |  | 10/10/2018 |
| Lab Reagent Blank | LRB1 | 0 | pg |  |  |  |  |  |  | 10/10/2018 |
| Lab Reagent Blank | LRB2 | 0.00501 | pg |  |  |  |  |  |  | 10/10/2018 |
| Matrix Spike | R740441B2 | 6,424.95 | ng/L | 5000 | 1,310.47 | 102.2897 | 71-125 |  |  | 10/10/2018 |
| Method Duplicate | R731531B2 | 5,095.60 | ng/L |  | 5,217.04 |  |  | 2.36 | 35 | 10/10/2018 |
| Ongoing Precision and Recovery | OPR1 | 9.68 | ng/L | 10 |  | 96.80894 | 67-133 |  |  | 10/10/2018 |
| Ongoing Precision and Recovery | OPR2 | 10.40 | ng/L | 10 |  | 103.8559 | 67-133 |  |  | 10/10/2018 |
| Standard Reference Material | NIST955c 1 | 4,148.27 | ng/L | 4500 |  | 92.1838 | 67-133 |  |  | 10/10/2018 |
| Lab Fortified Blank | LFB1 | 4,107.96 | ng/L | 5000 |  | 82.15929 | 67-133 |  |  | 10/31/2018 |
| Lab Reagent Blank | LRB1 | 0 | pg |  |  |  |  |  |  | 10/31/2018 |
| Lab Reagent Blank | LRB2 | 0 | pg |  |  |  |  |  |  | 10/31/2018 |
| Matrix Spike | N511531B1 | 6,064.55 | ng/L | 5000 | 946.1465 | 102.3681 | 71-125 |  |  | 10/31/2018 |
| Method Duplicate | N511691B1 | 4,596.52 | ng/L |  | 3,865.65 |  |  | 17.3 | 35 | 10/31/2018 |
| Ongoing Precision and Recovery | OPR1 | 9.60 | ng/L | 10 |  | 95.96401 | 67-133 |  |  | 10/31/2018 |
| Ongoing Precision and Recovery | OPR2 | 11.30 | ng/L | 10 |  | 112.8954 | 67-133 |  |  | 10/31/2018 |
| Standard Reference Material | NIST955c | 4,393.34 | ng/L | 4500 |  | 97.62967 | 67-133 |  |  | 10/31/2018 |
| Standard Reference Material | NIST955c | 4,023.13 | ng/L | 4500 |  | 89.40293 | 67-133 |  |  | 10/31/2018 |
| Lab Fortified Blank | LFB1 | 4,720.46 | ng/L | 5000 |  | 94.40923 | 67-133 |  |  | 11/2/2018 |
| Lab Reagent Blank | LRB1 | 0 | pg |  |  |  |  |  |  | 11/2/2018 |
| Lab Reagent Blank | LRB2 | 0 | pg |  |  |  |  |  |  | 11/2/2018 |
| Matrix Spike | N511921B1 | 6,461.34 | ng/L | 5000 | 1,369.76 | 101.8315 | 71-125 |  |  | 11/2/2018 |
| Method Duplicate | N511611B1 | 2,185.45 | ng/L |  | 1,748.99 |  |  | 22.2 | 35 | 11/2/2018 |
| Ongoing Precision and Recovery | OPR1 | 9.36 | ng/L | 10 |  | 93.61283 | 67-133 |  |  | 11/2/2018 |
| Ongoing Precision and Recovery | OPR2 | 9.66 | ng/L | 10 |  | 96.6371 | 67-133 |  |  | 11/2/2018 |
| Standard Reference Material | NIST955c | 3,815.31 | ng/L | 4500 |  | 84.78468 | 67-133 |  |  | 11/2/2018 |
| Standard Reference Material | NIST955c | 3,509.24 | ng/L | 4500 |  | 77.98303 | 67-133 |  |  | 11/2/2018 |
| Standard Reference Material | NIST955c | 5,516.26 | ng/L | 4500 |  | 122.5836 | 67-133 |  |  | 11/2/2018 |
| Lab Fortified Blank | LFB1 | 5,511.04 | ng/L | 5000 |  | 110.2208 | 67-133 |  |  | 11/5/2018 |
| Lab Reagent Blank | LRB1 | 0 | pg |  |  |  |  |  |  | 11/5/2018 |
| Lab Reagent Blank | LRB2 | 0 | pg |  |  |  |  |  |  | 11/5/2018 |
| Matrix Spike | I621141B1 | 34,020.15 | ng/L | 5000 | 29,656.48 | 87.27328 | 71-125 |  |  | 11/5/2018 |
| Method Duplicate | I621181B1 | 26,442.82 | ng/L |  | 32,556.05 |  |  | 20.7 | 35 | 11/5/2018 |
| Ongoing Precision and Recovery | OPR1 | 13 | ng/L | 10 |  | 129.6782 | 67-133 |  |  | 11/5/2018 |
| Ongoing Precision and Recovery | OPR2 | 10.3 | ng/L | 10 |  | 102.8945 | 67-133 |  |  | 11/5/2018 |
| Standard Reference Material | NIST955c | 3,859.17 | ng/L | 4500 |  | 85.75935 | 67-133 |  |  | 11/5/2018 |
| Standard Reference Material | NIST955c 1 | 4,849.42 | ng/L | 4500 |  | 107.7648 | 67-133 |  |  | 11/5/2018 |
| Lab Fortified Blank | LFB1 | 5,418.82 | ng/L | 5000 |  | 108.3763 | 67-133 |  |  | 11/6/2018 |
| Lab Reagent Blank | LRB1 | 0 | pg |  |  |  |  |  |  | 11/6/2018 |
| Lab Reagent Blank | LRB2 | 0 | pg |  |  |  |  |  |  | 11/6/2018 |
| Matrix Spike^a^ | I621221B1 | 48,921.37 | ng/L | 5000 | 49,516.60 | -11.9046 | 71-125 |  |  | 11/6/2018 |
| Method Duplicate | I620971B1 | 28,693.78 | ng/L |  | 31,784.31 |  |  | 10.2 | 35 | 11/6/2018 |
| Ongoing Precision and Recovery | OPR1 | 9.84 | ng/L | 10 |  | 98.44487 | 67-133 |  |  | 11/6/2018 |
| Ongoing Precision and Recovery | OPR2 | 9.73 | ng/L | 10 |  | 97.33715 | 67-133 |  |  | 11/6/2018 |
| Standard Reference Material | NIST955c 1 | 4,591.65 | ng/L | 4500 |  | 102.0367 | 67-133 |  |  | 11/6/2018 |
| Standard Reference Material | NIST955c | 4,530.70 | ng/L | 4500 |  | 100.6822 | 67-133 |  |  | 11/6/2018 |
| Lab Fortified Blank | LFB1 | 4,779.41 | ng/L | 5000 |  | 95.58819 | 67-133 |  |  | 11/7/2018 |
| Lab Reagent Blank | LRB1 | 0 | pg |  |  |  |  |  |  | 11/7/2018 |
| Lab Reagent Blank | LRB2 | 0.207 | pg |  |  |  |  |  |  | 11/7/2018 |
| Matrix Spike | I620651B2 | 15,735.05 | ng/L | 5000 | 9,853.08 | 117.6393 | 71-125 |  |  | 11/7/2018 |
| Matrix Spike | N511721B1 | 6,243.22 | ng/L | 5000 | 1,396.69 | 96.93061 | 71-125 |  |  | 11/7/2018 |
| Matrix Spike | I621221B1 | 63,111.74 | ng/L | 20000 | 43,919.24 | 95.96247 | 71-125 |  |  | 11/7/2018 |
| Method Duplicate | I620671B2 | 4,675.19 | ng/L |  | 4,718.99 |  |  | 0.932 | 35 | 11/7/2018 |
| Method Duplicate | N511721B1 | 1,548.05 | ng/L |  | 1,396.69 |  |  | 10.3 | 35 | 11/7/2018 |
| Ongoing Precision and Recovery | OPR1 | 9.62 | ng/L | 10 |  | 96.2278 | 67-133 |  |  | 11/7/2018 |
| Ongoing Precision and Recovery | OPR2 | 11.50 | ng/L | 10 |  | 114.8299 | 67-133 |  |  | 11/7/2018 |
| Ongoing Precision and Recovery | OPR3 | 9.53 | ng/L | 10 |  | 95.25057 | 67-133 |  |  | 11/7/2018 |
| Standard Reference Material | NIST955c | 4,593.64 | ng/L | 4500 |  | 102.0809 | 67-133 |  |  | 11/7/2018 |
| ^a^Spike level too low. RPD between source and matrix spike minus spike concentration is 11.98, well within the acceptable range for a replicate. Other batch QC support successful digestion without loss of analyte. Sample redigested with 4x spike concentration on 11/7/18 | | | | | | | | | | |
