# Supplementary material for: Exposure to total and methylmercury among pregnant women in Suriname: sources and public health implications
Source: J Expo Sci Environ Epidemiol. Author manuscript; Available in PMC 2021 Feb 1. (PMC7704553; doi:10.1038/s41370-020-0233-3)
Supplement: Supplementary File 4 [file NIHMS1599501-supplement-Supplementary_File_4.docx]

| Table S4. Quality assurance and quality control indicators for all analyses of total mercury in hair samples from research participants in Suriname. All QA/QC indicators are met regarding the laboratory methods used in this study. | | | | | | | | | | |
| --- | --- | --- | --- | --- | --- | --- | --- | --- | --- | --- |
| Type | Name/ID | Final Result | Units | Spike Concentration | Source Result | % REC | % REC Limit | RPD | RPD Limit | Analysis Date |
| Lab Fortified Blank | LFB | 943.3434 | µg/Kg | 1000 |  | 94.33434 | 77-123 |  |  | 8/28/2018 |
| Lab Reagent Blank | LRB1 | 3.07 | pg |  |  |  |  |  |  | 8/28/2018 |
| Lab Reagent Blank | LRB2 | 0.65 | pg |  |  |  |  |  |  | 8/28/2018 |
| Matrix Spike | I621001 | 22,427.59 | µg/Kg | 6031 | 16,217.81 | 102.9644 | 71-125 |  |  | 8/28/2018 |
| Matrix Spike | N511691 | 5,102.71 | µg/Kg | 2595 | 2,543.39 | 98.62524 | 71-125 |  |  | 8/28/2018 |
| Method Duplicate | I620531 | 2,609.87 | µg/Kg |  | 2,251.90 |  |  | 14.7 | 24 | 8/28/2018 |
| Method Duplicate | I620711 | 1,773.75 | µg/Kg |  | 1,655.98 |  |  | 6.87 | 24 | 8/28/2018 |
| Ongoing Precision and Recovery | OPR1 | 975.00 | µg/Kg | 1000 |  | 97.50552 | 77-123 |  |  | 8/28/2018 |
| Ongoing Precision and Recovery | OPR2 | 931.00 | µg/Kg | 1000 |  | 93.05399 | 77-123 |  |  | 8/28/2018 |
| Ongoing Precision and Recovery | OPR3 | 1,050.00 | µg/Kg | 1000 |  | 104.7498 | 77-123 |  |  | 8/28/2018 |
| Second Source Spike | QCS1 | 387.00 | µg/Kg | 400 |  | 96.63648 | 77-123 |  |  | 8/28/2018 |
| Standard Reference Material | BCR-397 | 11,739.28 | µg/Kg | 12300 |  | 95.44132 | 77-123 |  |  | 8/28/2018 |
| Standard Reference Material | APPLE LEAVES | 41.14729 | µg/Kg | 44 |  | 93.51657 | 77-123 |  |  | 8/28/2018 |
| Lab Fortified Blank | LFB | 858.6248 | µg/Kg | 1000 |  | 85.86248 | 77-123 |  |  | 8/30/2018 |
| Lab Reagent Blank | LRB1 | 0.69 | pg |  |  |  |  |  |  | 8/30/2018 |
| Lab Reagent Blank | LRB2 | 1.50 | pg |  |  |  |  |  |  | 8/30/2018 |
| Matrix Spike | R732801 | 1,726.79 | µg/Kg | 1713 | 42.51531 | 98.32319 | 71-125 |  |  | 8/30/2018 |
| Matrix Spike | R731611 | 2,378.64 | µg/Kg | 2334 | 200.2216 | 93.33419 | 71-125 |  |  | 8/30/2018 |
| Method Duplicate | R740441 | 451.9256 | µg/Kg |  | 376.1312 |  |  | 18.3 | 24 | 8/30/2018 |
| Method Duplicate | R732171 | 1,409.24 | µg/Kg |  | 1,401.55 |  |  | 0.547 | 24 | 8/30/2018 |
| Ongoing Precision and Recovery | OPR1 | 1,000 | µg/Kg | 1000 |  | 100.2028 | 77-123 |  |  | 8/30/2018 |
| Ongoing Precision and Recovery | OPR2 | 963 | µg/Kg | 1000 |  | 96.25934 | 77-123 |  |  | 8/30/2018 |
| Ongoing Precision and Recovery | OPR3 | 1,030 | µg/Kg | 1000 |  | 102.8713 | 77-123 |  |  | 8/30/2018 |
| Second Source Spike | QCS1 | 399 | µg/Kg | 400 |  | 99.85483 | 77-123 |  |  | 8/30/2018 |
| Standard Reference Material | BCR-397 | 11,449.52 | µg/Kg | 12300 |  | 93.08551 | 77-123 |  |  | 8/30/2018 |
| Standard Reference Material | APPLE LEAVES | 39.03151 | µg/Kg | 44 |  | 88.70797 | 77-123 |  |  | 8/30/2018 |
| Lab Fortified Blank | LFB | 980.87 | µg/Kg | 1000 |  | 98.087 | 77-123 |  |  | 9/11/2018 |
| Lab Reagent Blank | LRB1 | 0.00 | pg |  |  |  |  |  |  | 9/11/2018 |
| Lab Reagent Blank | LRB2 | 0.00 | pg |  |  |  |  |  |  | 9/11/2018 |
| Matrix Spike | R732111 | 2,660.19 | µg/Kg | 1466 | 1,333.17 | 90.51972 | 71-125 |  |  | 9/11/2018 |
| Matrix Spike | I620621 | 6,519.86 | µg/Kg | 2041 | 4,458.58 | 115.5524 | 71-125 |  |  | 9/11/2018 |
| Method Duplicate | N511321 | 2,231.79 | µg/Kg |  | 2,197.21 |  |  | 1.56 | 24 | 9/11/2018 |
| Ongoing Precision and Recovery | OPR1 | 966 | µg/Kg | 1000 |  | 96.6465 | 77-123 |  |  | 9/11/2018 |
| Ongoing Precision and Recovery | OPR2 | 916 | µg/Kg | 1000 |  | 91.63376 | 77-123 |  |  | 9/11/2018 |
| Ongoing Precision and Recovery | OPR3 | 1,010 | µg/Kg | 1000 |  | 100.625 | 77-123 |  |  | 9/11/2018 |
| Second Source Spike | QCS1 | 399 | µg/Kg | 400 |  | 99.65961 | 77-123 |  |  | 9/11/2018 |
| Standard Reference Material | BCR-397 | 12,617.59 | µg/Kg | 12300 |  | 102.582 | 77-123 |  |  | 9/11/2018 |
| Standard Reference Material | APPLE LEAVES | 38.78173 | µg/Kg | 44 |  | 88.1403 | 77-123 |  |  | 9/11/2018 |
| Lab Fortified Blank | LFB | 1,046.30 | µg/Kg | 1000 |  | 104.6302 | 77-123 |  |  | 9/14/2018 |
| Lab Reagent Blank | LRB1 | 0.00 | pg |  |  |  |  |  |  | 9/14/2018 |
| Lab Reagent Blank | LRB2 | 1.44 | pg |  |  |  |  |  |  | 9/14/2018 |
| Matrix Spike | I620541 | 4,263.74 | µg/Kg | 1944 | 2,224.35 | 104.907 | 71-125 |  |  | 9/14/2018 |
| Matrix Spike | I620771 | 4,811.21 | µg/Kg | 851 | 4,104.78 | 83.01096 | 71-125 |  |  | 9/14/2018 |
| Method Duplicate | R732421 | 1,234.83 | µg/Kg |  | 1,183.83 |  |  | 4.22 | 24 | 9/14/2018 |
| Method Duplicate | I620681 | 3,248.07 | µg/Kg |  | 3,276.59 |  |  | 0.874 | 24 | 9/14/2018 |
| Ongoing Precision and Recovery | OPR1 | 962 | µg/Kg | 1000 |  | 96.20013 | 77-123 |  |  | 9/14/2018 |
| Ongoing Precision and Recovery | OPR2 | 959 | µg/Kg | 1000 |  | 95.91818 | 77-123 |  |  | 9/14/2018 |
| Ongoing Precision and Recovery | OPR3 | 972 | µg/Kg | 1000 |  | 97.21431 | 77-123 |  |  | 9/14/2018 |
| Second Source Spike | QCS1 | 408 | µg/Kg | 400 |  | 102.0639 | 77-123 |  |  | 9/14/2018 |
| Standard Reference Material | BCR-397 | 12,205.41 | µg/Kg | 12300 |  | 99.23099 | 77-123 |  |  | 9/14/2018 |
| Standard Reference Material | APPLE LEAVES | 39.63655 | µg/Kg | 44 |  | 90.08307 | 77-123 |  |  | 9/14/2018 |
